# “Us” and “them”: collective identity-building of far-right movements in *Chemnitz* and “Querdenken”

**DOI:** 10.3389/fsoc.2025.1680879

**Published:** 2025-11-06

**Authors:** Anja Schmidt-Kleinert

**Affiliations:** Department of Political Science, Philipps-University Marburg, Marburg, Germany

**Keywords:** frame analysis, online communication, social movement, antisemitism, Germany

## Abstract

Far-right actors successfully mobilized supporters for protests in the city of Chemnitz, Germany, in the summer of 2018, triggered by the fatal stabbing of a German national and the subsequent arrest of two asylum seekers. At first glance, they applied familiar enemy constructions in their online communication on the event. However, a more detailed analysis showed that these “enemies” were not targeted randomly. In this paper, I address the question of how the collective far-right actors in two German case studies—*Chemnitz* and “Querdenken”—framed their online communication on Facebook to foster the process of the respective social movement's collective identity-building. In particular, I focus on the role that the construction of out-groups, or “enemies” plays for their collective identity-construction. I apply frame analysis. The findings show that diagnostic master frames of “enemy” outgroups and “crisis” prove essential components; besides, diagnostic frames are more or less the only frame dimension identified. As master frames they hint to a collectively shared knowledge across the two discursive events.

## 1 Introduction

Far-right collective actors broadly mobilized their supporters in the city of Chemnitz, Germany, in the summer of 2018 by successfully applying enemy images familiar to the “New Right”—refugees, Muslims, “the media”, political opponents—to mobilize “concerned citizens” that resonated with their followers on social networks (Pfahl-Traughber, 2019). Over time, previous research showed, the actors did not target these “enemies” randomly but discursively organized them around an antisemitic core that culminated in questioning “the government and the (current political) system” (Schmidt-Kleinert, 2021; 126). This antisemitic core consisted of a Schmittian friend-foe thinking, claiming that there was an alleged conspiracy of the out-groups identified against “the German people”, and, moreover, that these out-groups allegedly received orders from an abstract “enemy”, “puppet-masters”. Two years later, opponents of the restrictions that the German government had implemented as a reaction to the COVID-19 pandemic started to organize regular nation-wide protests. At first glance, the discursive construction of the events in *Chemnitz* (2018–19) and of “Querdenken”^1^ groups (since 2020) have little in common. The events of *Chemnitz* (2018) took place against the background of “Europe's migration crisis” (from 2014 onward), with local protests organized by collective far-right actors, and the speed and the extent to which far-right groups were able to mobilize and take to the streets for weeks surprised many. For “Querdenken” groups, the organization of continuous demonstrations nation-wide had initially been a reaction to the “Corona crisis”, i.e., state-implemented restrictions in the context of the COVID-19 pandemic. Yet, as I will show, the discursive framing of “Querdenken's” online communication reveals several similarities and continuities with *Chemnitz* in terms of how in- and out-groups are framed.

According to Benford and Snow (2000), for social movements to succeed, they must effectively manage the core tasks of recruiting new members, acquiring resources, maintaining communication with their members, and constructing a collective identity. The sustained mobilization of followers online to repeatedly participate in large demonstrations in both cases suggests that respective collective actors were successful in creating a collective identity of their social movements allowing commitment among their followers. In this paper, I approach the phenomenon from a perspective of collective identity-building through framing. I focus on the question of how the in-group (“us”) and out-group(s) (“them”) are framed in the online communication of the two cases of *Chemnitz* and “Querdenken”. Taking a comparative perspective, I also investigate similarities and differences between the social movements within and across the discursive events (master frames). I apply Benford and Snow's (2000) framing approach to the online communication of the two case studies at hand on Facebook.

I structured the paper as follows: I will first review the research on collective identity-building and the role of emotions in social movement studies as well as on far-right framing of in-group and out-group(s) in Germany and transnationally. Second, I will present my methodological approach and introduce the two case studies. Third, I will present my empirical findings and critically discuss them.

## 2 Collective identity and social movements

Raschke (1985) described social movements as

a mobilizing collective social actor that pursues the goal of bringing about, preventing or reversing fundamental social change with a certain continuity based on high symbolic integration and low role specification by means of variable forms of organization and action. (Raschke, 1985; 77, original in italics, author's translation)

In this sense, social movements are “interactively created social products” (Soeffner, 1992; 196, as cited in Rucht, 2023; 8) that need to create a sense of belonging and an experience of commonly shared values and goals for their members and followers (Rucht, 2023; 12) to maintain successful mobilization. Not surprisingly, the concept of collective identity in social-movement studies intersects with several key sociological theories. Theories of identity emphasize how social identities are shaped on the individual level through interaction with and recognition by others (Goffman, 1959) and on the collective level through the “historical and civilizational backgrounds of the respective societies” (Eisenstadt, 1998; 229) or social groups, through “(cultural) codes” and respective “power struggles” and “struggles over resources” (Eisenstadt, 1998; 234). Moreover, collective identity is linked to theories of social constructionism, according to which meanings and identities are not inherent but constructed through social processes. In this sense, collective identity is based on the shared knowledge of a particular collective about “us” and “them” (Berger and Luckmann, 1966).

Flesher Fominaya (2010) argued that, like a social movement, its collective identity is not a fixed concept but fluid and constructed through the ongoing interactions between individuals within the movement and “in relation to the field or context in which the movement exists” (Flesher Fominaya, 2010; 397). Collective identity building then involves struggles over inclusion and exclusion; and the process of boundary formation helps define a movement's members, distinguishing them from those outside the movement. Social movements build a cohesive sense of “us” vs. “them”, which is crucial for sustaining mobilization and action; by constructing a collective identity, social movements draw lines between those who belong and those who do not, thus defining both their respective internal solidarity and external political stance (Flesher Fominaya, 2010; Diani, 1992). Collective framing helps individuals perceive themselves as part of a larger movement and can influence both recruitment and participation. Furthermore, collective identities shape the strategies and tactics employed by movements, aligning them with their overarching values and goals. Literature on social movements identified aspects of constructing a shared sense of inequality or injustice, establishing a common enemy, and forcing third parties in the conflict to take sides as central of collective identity (Daphi, 2011; 16). Besides, Flesher Fominaya (2010) emphasized the emotional dimension of collective identity, underscoring its crucial role in fostering solidarity among participants. Polletta and Jasper (2001) added that movements develop a collective identity through the articulation of shared grievances and aspirations that resonate with potential participants. Emotional engagement hence is essential for long-term commitment to a movement, as individuals” personal identities become intertwined with the movement's goals and values (see also Melucci, 1995).

## 3 Far-right major frames

Previous research has elaborated on various frames applied by far-right collective actors in Germany and transnationally. Major frames identified deal with “crisis”, “the people”, “the establishment”, “migration”, and “conspiracy”.

### 3.1 The “crisis”-frame

Moffitt (2015) elaborated on his hypothesis that the performance of crisis is “an internal core feature of populism”—or major frame—and develops a respective “model of the populist “performance” of crisis” (2015; 198). In this context, “crisis” was defined as something that is perceived—in other words, framed—as such by populist collective actors (cf. 2015; 197).

### 3.2 The “people” v “the establishment”-frame

Using documents published by Italian and German far-right collective actors as a data basis, Caiani and della Porta focused in a comparative case study on the question of “how the central populist frame (namely the people vs. the elite) is linked to the extreme right definition of the ‘us' and the ‘them”' (Caiani and della Porta, 2011; 180). They identified content and context of the application of major frames, such as “the people”, “the politicians”, the “far-right as an in-group” as subject actors, object actors and associated actions and adjectives in the analyzed documents. Particularly interesting in the context of the present study are the findings on “the people” as associated with passivity, “betrayal” and “robbed”, who “should wake up” and “take back its power” (2011; 191).

Taking another German far-right collective actor, “PEGIDA”^2^, as a case study, Volk identified two major frames that apply to “populism's antagonistic logic” (2023, 538): “the people” as a “democratic” in-group (Volk, 2023; 540f) against which the “political establishment” is constructed as a “totalitarian” out-group (Volk, 2023, 534). In this context, Volk showed, the far-right group also adapted the idea of leading a “peaceful revolution” (Volk, 2023, 543). Volk and Weisskircher (2023) elaborated on these frames in more detail. In a further empirical study, “the people” were also defined as a primordial “Volk” (e.g., Ahmed and Pisoiu, 2021).

### 3.3 The “migration”-frame

Frames of “crisis” and “the people” have often been linked to “migration” or “refugees” as a problem. Ahmed and Pisoiu (2021) showed how these frames were discursively linked by far-right groups to discursive strands of “Überfremdung” (“over-foreignization”), and migrants as “stabbers” or “rapists” (see also Closmann, 2020). Already the “bourgeois” authors of the “Heidelberg Manifesto of 17 June 1981” (ZEIT Archiv, 1982) denounced “Überfremdung” and blamed the migration policy of the then federal government for an alleged threat to “their own culture”.

“Islam” in this context has often been framed by far-right collective actors as (cultural) “other”, also in terms of “emotional framing” (Evolvi, 2019). She found “that Twitter users expressed anti-Muslim feelings in tweets involving anger, fear, hate, pride, sarcasm” (2019, 888).

### 3.4 The “conspiracy”-frame

Against the background that various conspiracy myths have been heavily shared during the Covid-19 pandemic, researchers also examined far-right framing in this context. Conspiracy myths are particularly interesting because not only far-right groups apply them, but they exist in all parts of society globally. Polta (2023) examined the link between conspiracy myths, antisemitism and antifeminism during the pandemic in three German-speaking Telegram channels. He identified several frames that were notably established in the context of Covid-19: “Bill Gates” and “chipping humans”, “the government as a marionette”, “pedophilia” and “QAnon”^3^. In addition, Bleakley (2023) showed how the “QAnon” conspiracy myth was distributed and established in far-right forums on Twitter.

### 3.5 Social media as a communication tool

Additionally, with time, far-right groups have made extensive use of social networks as a new aspect and form of social relationships and by now are well presented on various social networks. Social networks offer a high potential for mobilization purposes, since they are easy to use and allow direct communication with (potential) followers, vastly expanding the potential reach of groups and organizations. Kakavand (2024) also stressed the importance of social platforms for far-right actors: “especially social media are essential communication tools for the far right because they facilitate exchange between like-minded actors […]” (p. 38). Against this background, (Merrill and Åkerlund 2018) investigated particularly how Facebook's architecture enhances the distribution of racist discourse; Jacobs and Spierings (2019) did the same for Twitter. Yet, as the comparative study conducted by Klein and Muis (2019) showed, online-activism of far-right parties is usually based on limited political opportunities offline. The study found that if those parties have enough resources here to engage with potential supporters, one rather finds non-organizational far-right groups present on social media platforms.

Very broadly, already Krämer's study confirmed the use of different social network platforms by far-right groups to “circumvent[…] the traditional media” and to establish a direct relationship with their followers online on the one hand, and on the other hand to distribute their ideology, including an alleged representation of “the people” by far-right groups and their leaders or the construction and discursive exclusion of out-groups (Krämer, 2017). Yet, Gagnon observed still in 2020 that

[m]ost […] studies, however, focus on far-right groups' use of online platforms such as Web sites, blogs, and forums […]. As such, little is known about how far-right groups exploit some of the most widely used social media platforms such as Facebook, Twitter, and YouTube. (Gagnon, 2020; 358)

The present study contributes to further understand far-right framing of the Covid-19 pandemic, but also to place the findings into a broader analysis of the development of far-right master frames of out-groups as a repertoire for mobilization in Germany. To this aim, I analyzed the communication of four collective actors in two German case studies in public groups on Facebook.

## 4 Methodology

I conducted frame analysis of two cases studies, *Chemnitz* and “Querdenken”. For the present study, I first screened German media coverage of *Chemnitz* and “Querdenken” to identify major collective actors for mobilization in social networks online^4^. Media reports were used to inquire which collective actors appeared in the events surrounding far-right marches and pro-democratic civil society demonstrations against the right. The collective actors observed in the context of *Chemnitz* were explicitly linked to the organized far right in Germany; “Querdenken” groups had at least shown a certain openness toward the far right in terms of ideology, supporters” and background. Against this background, I decided to collect data only from the initial 12 months after the first “Querdenken” groups appeared since I was interested to investigate how the social movement framed its actions with time, giving them time to build a collective identity.

Due to the very large overall number of posts, I had to limit the text corpus. Since my focus was on how collective far-right actors framed their online-communication to mobilize supporters to the streets in the context of the discursive events, I considered when and to what extent they had entered the discourse. Finally, I chose four collective actors (two for *Chemnitz* and two for “Querdenken”). The database consists of selected online communication in public groups on Facebook; these were systematically archived as screenshots and saved as PDF files in real-time. Besides, since I ran a secondary analysis of data on *Chemnitz* collected in the context of a previous study (Schmidt-Kleinert, 2021), I also collected original data from public “Querdenken” groups on Facebook for the present study. I deliberately chose public groups instead of the rather restrictive groups or channels regarding visibility on Telegram and other social networks. But Facebook as a social platform also matched the needs of its users, and thus potential followers of far-right collective actors, in terms of connectivity (cf. Kakavand, 2024; Evans et al., 2017). Moreover, Kakavand explicitly mentioned far-right groups' use of Facebook as an organizing tool (2024; 44) and to reach wide-scale user engagement (ibid; 45). In this context, I argue that the possibility of establishing a public group on Facebook—in contrast to rather closed groups, e.g., on Telegram, that require access by a moderator and thus have a clandestine touch—allows far-right groups to distribute their messages like “mainstream” content and give them a legal, hence innocent, appearance.

The unit of analysis was the individual post. In a first step, I examined the texts for discourse strands and intersections between them based on critical discourse analysis (Jäger, 2015). To this end, I coded the material inductively, i.e., without predetermined analytical categories, and then formed conceptual categories, as suggested in the grounded theory approach (Glaser and Strauss, 2010). As an interim result of the coding process, it can be noted that in-group solidarity, enemy constructions and the legitimization of violence emerged as central issues. In a second step, I applied frame analysis as a sensitizing concept to the conceptual categories I had identified in the coding phase to make statements about how framing was used by the collective actors at hand to construct a collective identity in the process of their mobilization efforts^5^. The frames were analyzed inductively, and I used the conceptual categories identified in the first step (see above) as a basis to structure my empirical findings. It was only in a further step that I compared my findings with findings from previous research on far-right framing. I did so to keep the analytical process open to the exploration of new frames instead of merely confirming previous studies.

### 4.1 Frame analysis as a sensitizing concept

According to Benford and Snow, framing processes ultimately contribute to the collective identity construction of a social movement (2000; 614). Social movements create frames, conceptualized as “specific elements” of ideology (Rucht, 2023; 125), to communicate their “diagnosis of present problems” and a “solution or vision of a better world” (Rucht, 2023; 47). In this understanding, frames are defined as “action-oriented sets of belief and meanings to inspire and legitimize […] activities […] of the movement” (Benford and Snow, 2000; 614); they are “modes of interpretation […]” (Benford and Snow, 2000; 615). Frames add clarity and focus to a social movement's communication of ideology by placing specific aspects in the foreground of discussion (Daphi, 2011). Frame analysis therefore aims to deconstruct the processes that lead to and uphold collective identity in this context. Benford and Snow (2000) target “core framing tasks” (Benford and Snow, 2000; 615) that social movements must manage, which link to Daphi's core aspects of collective identity construction: injustice, enemies, and taking sides (see above). They distinguish between diagnostic, prognostic, and motivational tasks of framing: diagnostic frames are centered around the “amplif[ication of] victimhood” (Benford and Hunt, 1992; 39) and, consecutively, around the identification of who is to blame for the perceived inequality and can be presented as the “source of causality” (Benford and Snow, 2000; 616). Often, diagnostic frames are injustice frames (Benford and Snow, 2000; 615). Analytical dimensions of diagnostic frames include the identification of a problem, a mode of feeling related to a specific form of perceived unjustness, the attribution to specific others a source of responsibility, the identification of culpable agents, boundary-making, or a clear opposition of good and evil (Benford and Snow, 2000; 616). Prognostic tasks of framing refer to planned actions and the motivation of members and followers based on a presented solution to the most central problems communicated in a social movement's diagnostic frames. Analytical dimensions of prognostic frames contain a solution presented for the specific problem, e.g., in the form of a plan (Benford and Snow, 2000). Finally, motivational tasks of framing are a “call to arms”; they demand “appropriate vocabularies of motive”, namely severity, urgency, efficacy, and propriety (Benford and Snow, 2000; 617).

With a focus on collective identity-building of social movements, Lindekilde (2014) added identity frames that refer to the “us”, or, in-group, and oppositional frames, that help to construct “them”, or the out-group(s). In practice, Lindekilde's and Benford's and Snow's analytical dimensions overlap. Moreover, I paid particular attention to master frames. I follow here Swart's reconceptualization of “master frames as general symbolic frames that are culturally resonant to their historical milieux” (Swart, 1995; 466). As such, master frames connect different collective-action frames across social movements, in other words, across collective actors and/or across events (Benford and Snow, 2000) (see Table 1).

**Table 1:** Analytical dimensions of framing tasks derived from:

**Benford and Snow, 2000; Daphi, 2011**	**Lindekilde, 2014**	**Swart, 1995; Benford and Snow, 2000**	**Polletta and Jasper, 2001; Flesher Fominaya, 2010**
**Diagnostic frames**	**Identity frames**	**Master frames**	**Emotions**
Identification of a problem	Perceptions of “us”	Linking different frames	Emotional evaluation of an event
Mode of feeling unjust		a) Across events b) Across collective actors	Call to followers' emotions
Attribution of a source of responsibility, Culpable agents			
Boundary-making			Use of negative and/or positive emotions to define “us” and “them”
**Prognostic frames**	**Oppositional frames**		
Solution for a communicated problem	Perceptions of “them”		
Presentation of a plan			
**Motivational frames**			
Severity of action			
Urgency of action			
Efficacy of the presented plan			
Propriety			

As outlined, Benford and Snow (2000) explicitly refer to aspects of a shared sense of inequality or injustice, the construction of a common enemy, and forcing third parties to take sides in the conflict as discussed in the literature. Yet, further aspects, most importantly, the emotional attachment of members, a feeling of solidarity or the gradual construction of collective identity, remain outside their framework. Based on the work of Polletta and Jasper (2001) and Flesher Fominaya (2010), in my analysis, I operationalized emotional attachment as an emotional evaluation of events, calling to followers” emotions, as well as using negative or positive emotions to define “us” and “them” (see Table 1).

### 4.2 The case studies

On the last weekend of August 2018, the city of Chemnitz in Saxony (Eastern Germany) held its annual city festival. On Saturday evening, a young German citizen, Daniel H., died in the city's center (Brichzin et al., 2022; 13), and an arrest warrant was issued against two asylum seekers that were suspects in the death. In the following 2 weeks, local and national representatives of the organized far right in Germany called for a “funeral march” and for protests via their Facebook accounts. Björn Höcke, a key representative of the far-right party Alternative for Germany (AfD) and chairman of the party's state association of Thuringia, who has been monitored by the German Federal Office for the Protection of the Constitution since 2021 (Bundesamt für Verfassungsschutz, 2025), reacted early. He is a public figure of the far right and reached followers nationwide with his comments on this “violent incident”. The local far-right group “Bürgerbewegung Pro Chemnitz” continued after Höcke, also turning to other issues, and called for demonstrations, first for the duration of almost 2 weeks and later also for the occasion of the first “anniversary” of the incident in August 2019. Both Höcke and “Pro Chemnitz” were highly successful in their online mobilization via Facebook. In the following days, thousands of their supporters filled the city's streets, accompanied by many “ordinary citizens”. At the same time, various pro-democratic counter protests and a music festival aimed at showing solidarity with refugees and immigrants took place.

About 1.5 years later, several groups emerged under the label of “Querdenken” that protested measures taken by the German government as a reaction to the COVID-19 pandemic. Founded in April 2020 in Stuttgart, Baden-Württemberg, this movement had 80 to 120 local branches during its peak and organized regular marches in cities such as Stuttgart or Berlin. Since 2022, the German Federal Office for the Protection of the Constitution has monitored “Querdenken” protests, not necessarily classified as far right but understood as a new form of “anti-constitutional delegitimization of the state” (Götschenberg, 2022). Members have become more professionalized in their organization of events and communication on social networks. A group called “Querdenken 711” was founded during the first lockdown in Germany in April 2020, and many more local groups like it appeared on Facebook (usually as public groups) and/or Telegram (usually as closed groups) soon after. They all used the template of “Querdenken” in combination with the local landline area code as regional identifiers for their group names. By 2021, Facebook had closed many such groups, after parts of “Querdenken” had come into the focus of security agencies (Der Tagesspiegel Online, 2021; Sundermeyer, 2020). Members come from different social milieus, but what they have in common is their denial of or skepticism about the existence of the COVID-19 pandemic as well as their opposition to measures that were implemented with the intention of containing the spread of the virus; this often includes the perception that people have been coerced to get vaccinated (Nachtwey and Frey, 2021; Virchow et al., 2020). In detail, I added the following data to my corpus:

a) for *Chemnitz*, the homicide of Daniel H. on the weekend of the Chemnitz city festival 2018 and the following about two weeks of vigorous mobilization activities on Facebook as well as marches, counter demonstrations, and the #wirsindmehr solidarity concert on 3 September 2018. Björn Höcke reacted as a public figure from the far-right spectrum to the violent incident of 26 August 2018, while the “Bürgerbewegung Pro Chemnitz” (“Pro Chemnitz Citizens' Movement”) is a relevant collective actor directly at the site of the violent incident due to its local roots. Both Höcke and the “Bürgerbewegung Pro Chemnitz” significantly promoted mobilization on the streets by initiating a “mourning march” and further demonstrations. Höcke withdrew shortly after the #wirsindmehr-concert. “Pro Chemnitz” got even more active and published posts on the topic very once to several times per day for a week. After 10 September 2018, “Pro Chemnitz” stopped posting on the discursive event. A second survey point was set for the end of the trial against one of the defendants for the death of Daniel H., with selected starting points such as the publicly accessible Facebook pages and Facebook groups of “Bürgerbewegung Pro Chemnitz” and AfD Chemnitz. The verdict was announced on 22 August 2019, just a few days before the anniversary of the marches at the end of August. Data were collected manually between 26 August 2018, and 10 September 2018; the second survey phase covered the period of 21 August to 26 August 2019 (Höcke: 8, “Pro Chemnitz”: 15 protest-related posts). Table 2 lists the dates of the individual posts considered for analysis.
b) Regarding “Querdenken” groups, I focused on “Querdenken 711”, the one that emerged first, established in Stuttgart (Baden Württemberg), and on “Querdenken 30”, the group local to Berlin. In Berlin, a large protest at the end of August 2020 culminated in an attempt by some participants to storm the Reichstag building. I started to follow the online communication of both public groups in May 2021, when the first posts to hint at plans for a central protest in Berlin on the anniversary of the 2020 assault on the Reichstag started to appear. I stopped data collection after that protest had taken place (25 August 2021). I covered the online communication of the two “Querdenken” groups at hand in the period of 30 April to 31 August 2021. Protest-related posts culminated appr. 4 weeks before the announced demonstration on 25 August 2021, in Berlin. In the analysis, I considered only posts I coded as collective framing or protest-related, which included all posts that asked e.g., for financial donations to the movement (“Querdenken 030”: 3, “Querdenken 711”: 15 protest-related posts). Table 3 lists the dates of the individual posts considered for analysis.

**Table 2:** Overview of analyzed Facebook posts on *Chemnitz*.

**Public group name: Björn Höcke**	**Public group name: “Bürgerbewegung Pro Chemnitz”**
26 August to 10 September, 2018, and 26-30 August, 2019: Total of 8 posts	26 August to 10 September, 2018, and 26-30 August, 2019: Total of 15 posts
1 Facebook post on 27 August, 2018.	1 Facebook post on 3 September, 2018.
1 Facebook post on 28 August, 2018.	2 Facebook posts on 4 September, 2018.
2 profile picture changes on 30 August, 2018.	1 Facebook post on 5 September, 2018.
2 Facebook posts on 31 August, 2018.	3 Facebook posts on 6 September, 2018.
1 Facebook post on 1 September, 2018.	5 Facebook posts on 7 September, 2018.
1 Facebook post on 2 September, 2018.	2 Facebook posts on 8 September, 2018.
	1 Facebook post on 9 September, 2018.
	1 Facebook-post on 26 September, 2019

**Table 3:** Overview of analyzed Facebook posts on “Querdenken”.

**Public group name: “Querdenken 030”**	**Public group name: “Querdenken 711”**
1 May to 25 August, 2021: Total of 10 posts, of which 3 were of topic on collective identity-building and included in the analysis	1 May to 25 August, 2021: Total of 200 posts, of which 15 were of topic on collective identity-building and included in the analysis
1 Facebook post on 1 May, 2021.	1 Facebook post on 3 August, 2021.
1 Facebook post on 18 July, 2021.	2 Facebook posts on 4 August, 2021.
1 Facebook post on 22 August, 2021.	1 Facebook retweet on 6 August, 2021.
	2 Facebook post on 10 August, 2021.
	1 Facebook post on 13 August, 2021.
	2 Facebook posts on 14 August, 2021.
	3 Facebook posts on 19 August, 2021.
	2 Facebook posts on 20 August, 2021.
	1 Facebook post on 23 August, 2021.

In the following, I will elaborate on how the collective actors of these two case studies framed their online communication with a particular focus on their construction of a collective identity.

## 5 “Them” and “us” in *Chemnitz* and “Querdenken” posts

In the online communication on *Chemnitz* and of “Querdenken”, a great effort becomes visible to frame problems in specific ways and to position group members as victims of these problems in German society. The collective actors at hand identify three major problems that serve as the basis for their framing: a “crisis”, an antagonistic group of “elites”, and an opposition between “the people” and “puppet masters”.

Based on their identification of central problems, Höcke, “Pro Chemnitz”, “Querdenken711” and “Querdenken 030” provide a clear idea of who is to blame for them and construct common “enemies”. Few frames in this context are event-specific; most of the frames applied can be identified as master frames. Both, specific and master frames, present the image of moving toward a catastrophe, as shortly after the respective trigger events, the collective actors in both cases refer to a mode of “crises”.

### 5.1 Diagnostic master frame: “elites”

In one of his first posts in the context of *Chemnitz*, on 28 August 2018, Höcke claims a “lack of grief”, in *Chemnitz* and nation-wide, in response to the death of Daniel H. (the trigger event in this case). He appeals to the emotions of his readers, “pity” and “grief”, exploiting these reactions to formulate a political demand—in his words, a rational consequence that shows “awareness”: “rage” against those “who are responsible for this”. By doing so, Höcke reassures all those who might be reluctant to send a “visible signal” that “showing anger is human”. He, together with two other chairmen of the AfD's state associations of Saxony and Brandenburg, calls for a “funeral march” that should be “peaceful and solemn”. Höcke strikes a solemn pose in both tone and image: the accompanying black-and-white photograph shows him thoughtful, holding a white rose. Yet, simultaneously, he calls for “bundling our anger into a clearly visible signal”—effectively counteracting his own appeal to solemnity.

In line with their diagnosis of the government as incapable of managing the alleged “ongoing crisis”, an antagonism in the form of the government or “elites” that stands in opposition to “the people” and is to blame for that crisis is constructed. This frame is not unique to *Chemnitz* but has long been used by far-right actors (Pfahl-Traughber, 2019; Mudde and Kaltwasser, 2017). Höcke runs a latent trace of this antagonism through his entire text; he writes about “us” vs. “them” in his posts in the context of the “funeral march”, following his statement of a lack of grief:

But what goes through our minds when we realize that this person could still be alive? When we realize that he is the next avoidable victim of irresponsible government policy that cold-bloodedly accepts the multiple deaths of locals? A policy that fanatically pursues a migration agenda contrary to the law and the majority opinion of the population, denying, concealing, or downplaying all the ugly side effects of illegal mass immigration? (Höcke, 28 August 2018)

Höcke contrasts features of his in-group against those of “them”: “compassionate” vs. “ice-cold”, “victims” vs. “perpetrators”, “natives” vs. “illegal mass immigration”, “majority opinion of the population” vs. “politicians”, “reason” vs. “madness”, “rule of law” vs. “fanatics”, “peaceful protesters” vs. “provocateurs”, “our country” vs. “forced multiculturalism” (28 August 2018). By doing so, Höcke moves from the concrete trigger event to a broader discussion of a fundamental gap between “ordinary citizens” and “elites”, including the idea that there is a powerful minority that rules and works against “the people”, who represent the “majority”.

“Pro Chemnitz” also contribute to the antagonistic relationship between “elites” and “the people”, e.g., by posting a “poem” (26 August 2019) that perpetuates a main theme from modern “New Right” propaganda: the opposition between “elites” and “the people” as well as the idea that these elites are controlled by a third, hidden entity (in the form of various conspiracy myths), which will ultimately take over and be in complete control (Pfahl-Traughber, 2019). In terms of language, the poem appears to be much older than it is (Stoppt die Rechten, 2018) and therefore takes on prophetic traits that help to mobilize supporters, such as for the marches on the homicide's anniversary.

### 5.2 Diagnostic frame: “war of cultures”—“Pro Chemnitz”

“Pro Chemnitz” develop the thread of “us” vs. “them” further. Readers are sworn into a “culture war”, which is even turned into a hashtag (“#Kulturkampf”, posts of 5–6 September 2018). “Pro Chemnitz” frame the alleged “war” as between “us”, i.e., “the people”, and “them”, i.e., “a cartel” that consists of the government, media, and opponents on the political left, and offering a more concrete interpretation of “them”.

Again, they do not identify such a war between “locals” and “refugees”, as the trigger event might suggest (see above). The “real enemies”, in line with “New-Right” thinking (Weiß, 2017), are not “asylum seekers”, who merely served as a trigger for *Chemnitz*, but the democratic political system that has allowed “them” to invade “our” space and has led to an abandonment of “our own” culture. Weiß's analysis shows, referring to a larger context, that a decisive component for identity formation on the far right is the cultural dimension of a friend-foe dichotomy, a concept borrowed from Carl Schmitt (Weiß, 2017). In this reading, representatives of the far right propagate a “moral annihilation of one's “own” culture” by “Americanism” in the “Kulturkampf” they conjure up (Weiß, 2017, 216), “which appears as a friend, but […] is fundamentally different from one's ‘own”' (Weiß, 2017; 213). The “real enemies”, thus, disguise their actions and act in the background.

Elaborating on the “war” frame, “Pro Chemnitz” also adds to the interconnection of the master frames of “elites”, “cartel media”, and “puppet masters” (which I will discuss in more detail below).

### 5.3 Diagnostic master frame: “rule of law”—“Querdenken”

In the context of various events during the COVID-19 pandemic, it was not only collective actors of the far right that repeatedly brought up Article 20, section 4 of the German Constitution, claiming that it guaranteed a right to resistance that legitimized their actions (e.g., “Querdenken 711”, post 1 of 19 August 2021). Though this is another master frame of the far right, not all collective actors in the two case studies touched this issue. Yet, “Querdenken” groups adapted it and stressed that their basic rights were endangered by government restrictions and that official COVID-19 restrictions broke with the “rule of law” (“Querdenken 711”, post 1 on 14 August 2021).

A central theme in this regard is that of “freedom”. On 20 August 2021 (post 1), “Querdenken 711” call for a “Day of Freedom”, stating: “We demand the immediate restoration of all basic rights, an immediate halt to the discrimination & oppression of minorities based on their beliefs & exercise of their freedom of expression”. Yet, as the statement goes on, it becomes clear that the group does not think of racial, religious, or gendered minorities that face discrimination in German society but instead regard themselves as a “minority” that needs protection: “We stand for bodily self-determination & a free vaccination decision” (“Querdenken 711”, post 1 on 14 August 2021). Their definition of “freedom” puts individual freedom over collective solidarity (Nachtwey and Frey, 2021), although many of their posts mentioning “freedom” suggest otherwise by claiming to care “for the weakest in our society”:

“[…] people who want to LIVE... and not just SURVIVE. Who stand up for each other and work together for peaceful change. Who stand up for freedom, democracy, the rule of law, and an unconditional, free VACCINATION DECISION for ALL people and show their faces. For themselves, but also for the weakest in our society.” (“Querdenken 30” on 18 July 2021, emphasis in the original)

### 5.4 Diagnostic master frame: “crisis” and the “government's mishandling”

The collective actors offer the identification of a “crisis” as a general diagnosis of our current times. The actual “crisis” constructed is interchangeable and flexible so it can be adapted based on specific trigger events, but it is always “elites” that are made responsible for the respective crisis presented. In this context, the collective actors reproduce “established” far-right slogans, e.g., these include “Chemnitz is everywhere” (“Pro Chemnitz” on 9 September 2018), which bears a deliberate similarity to “Kandel is everywhere” (Closmann, 2020; the slogan references the murder of a German girl by her Afghan refugee ex-boyfriend in Kandel; Germany, in December 2017). By doing so, in both case studies, collective actors aim to produce a feeling of an ongoing state of crisis that will ultimately lead to complete destruction, one of the master frames of the “New Right” (Schilk, 2024; Mudde and Kaltwasser, 2017; Moffitt, 2015). As a pattern, followers are indirectly reminded of modes of “crises” in other frames applied, e.g., when speaking of “attacks”, “fanatics”, “war”, or “being suppressed” to make them feel threatened and fearful.

These feelings are reinforced by the fact that authorities are constantly presented as weak and unable to manage the circumstances, as another strategy to mobilize their supporters. For instance, in both case studies, collective actors directly attack the government for its management of a “crisis”, be it “migration” (*Chemnitz*) or “Corona” (“Querdenken”). In this context, Höcke, for instance, speaks of a “migration agenda”, a hidden plan, of the government (Höcke on 28 August 2018), connecting the master frame of “crisis” to the master frames of blaming the “elites” and “puppet masters” (see below).

### 5.5 Diagnostic master frame: “cartel media”

Höcke introduces the frame of a “cartel” over the span of several posts. Following his dramaturgy of adding on *Chemnitz* over time, he, in his very first (27 August 2018), accuses “the media” of not reporting truthfully because they wrote about the marches of the far right after the “violent incident” instead “about the actual victims”. Höcke leaves vague who these “actual victims” and the perpetrators are in his view and thus leaves room for interpretation, in which his followers engage in their comments to the posts. The day after (post 2 on 28 August 2018), he claims that “the media” belong to a “cartel”, without elaborating further, followed by a claim that there is “downright a smear campaign against Chemnitz”, i.e., against “ordinary citizens” (1 September 2018). In the latter, Höcke finally shortly elaborates on the “cartel” and the connections he sees between “leading media”, “Antifa Twitter accounts” and “(then) government spokesperson (Steffen) Seibert”. By speaking of a “cartel”, he also introduces aspects of a conspiracy, linking his take to the master frame of “puppet masters” (see below).

“Pro Chemnitz”, again, take up Höcke's thread and accuse “the media” of contributing to the government's attempt of distraction. They become more concrete than Höcke, discussing examples to substantiate their claims, such as complaining about the media's reporting on Nazi salutes at demonstrations rather than on “the murder” (8 September 2018, post 1). “Pro Chemnitz” also suggest that “the media” is allowed to write anything: “you just have to show or even just say that you are on the “right” side (i.e., not ours), and then you can get away with practically anything. #Kulturkampf”, (5 September 2018), adding to Höcke's claim of a “cartel” as an enemy that casts “the people” as “wrong”. On the next day, they add:

“UNEDITED LIVE FOOTAGE. WHAT THE MEDIA IS HIDING FROM US... […] Antifa storms to the front of the march and attempts to attack AfD politicians!” (6 September 2018, emphasis in the original).

Three years later, “Querdenken 711” add to the enemy construct of “cartel media” by likening alleged “anti-democratic” attitudes of journalists to the Nazi propaganda publication *Der Stürmer*:

“Press release: Reichstag storming propaganda at Stürmer level Stuttgart/August 4, 2021 Public broadcasters and other media outlets continue to claim that the citizens' movement Querdenken was responsible for the event at the Reichstag on August 29, 2020, and for storming the steps.” (4 August 2021, post 2).

### 5.6 Diagnostic master frame: “puppet masters” and “marionettes”

In an adjacent frame to that of the “cartel”, the collective actors introduce a greater conspiracy, one led by “puppet masters”. The function of conspiracy myths is to offer simple explanations for complex relationships; what is not understood on the surface is often attributed to “string-pullers acting in the background” and their secret plans. As such, conspiracy myths are an integral part of an antisemitic world view, in which the “destructive” element for one's own way of life (e.g., in a “culture war”, see above) is particularly emphasized (Salzborn, 2020; Lipowsky, 2020). Against the backdrop of a “Jewish foreign body” as a surface of negative projection, conspiracy myths contribute to the creation of one's own positive identity in opposition. Hence, it is through the deconstruction of conspiracy myths that antisemitic rhetorical patterns are made visible in the present analysis (Salzborn, 2020).

Höcke contributes to the conspiracy frame with every post he publishes on *Chemnitz*. The “violent incident” serves as the trigger event, but the focus of his posts immediately switches to “common enemies”—false media reports, political opponents allegedly paid by the government, and the government as incapable of managing crises. Finally, a conspiracy is suggested, a “cartel” that pursues an “agenda”, e.g., to assert “forced multiculturalism”. Thus, over the course of the events, he is devoted to a dramaturgic strategy of escalation.

“Querdenken 711” (4 August 2021, post 2) add to the hierarchy of “enemies” established in *Chemnitz*. In their rhetoric, “the people” are standing up for their rights, even if the political elites lie (“Querdenken 711”, 3 August 2021). They also openly engage in the reproduction of conspiracy myths of an alleged worldwide Jewish conspiracy (whose “leaders” are often framed as “puppet masters”): followers are presented with a direct reference to “QAnon” (“there's so many still asleep”) in a “Querdenken 711” post on 19 August 2021 and can read that “in order to wake them up, the lies must be exposed, the puppet masters must be exposed”. In this context, the concept of “awakening” features strongly (Lipowsky, 2020). On 10 August 2021 (2), “Querdenken 711” retweet a video, suggesting a “View Behind the Matrix on Odyssey”, which refers to the US-American movie *The Matrix*, which is particularly famous among male supremacy “Incel” groups (Kelly et al., 2021). On 14 August 2021, the group retweets another video posted by “Anonymous Schweiz”:

In this video, however, we show you the importance of [… the Tavistock] institute, because as harmless as it may sound, there is now no aspect of life on which it does not have influence. The goal is to manipulate public opinion and steer it in the desired direction. The institute was financed by the Rockefeller Foundation. (Querdenken 711, 14 August 2021, post 2)

Again, this post contributes to an alleged conspiracy, as obviously very few people—in fact, only those who have been “awakened”—know about the institute. This framing is strengthened by adding information about a donor, the “Rockefeller Foundation”, which refers to “East Coast capital”, and thus another antisemitic stereotype (Knappertsbusch, 2016). In this context, previous studies on “Querdenken” have shown the application of conspiracy myths as a driving force of mobilization in their online communication (Nachtwey and Frey, 2021).

The enemy images constructed in the online communication on *Chemnitz* and “Querdenken” continue to serve conspiracy myths of modern antisemitism (Langer, 2022): the abstract, “real” enemy of “the Jew” materializes here into common, “visible enemies” that are closer in reach—the “media”, “Antifa” groups, and political “elites”.

### 5.7 Prognostic master frame: “peaceful resistance”

In *Chemnitz*, there is a clear division of labor for the mobilization of protesters: Höcke creates the discursive event by commenting on a local incident, locates responsibility in authorities, and uses conspiracy myths to introduce a common enemy for his followers to blame. “Pro Chemnitz” repeat his threads, and sometimes make them more concrete, better “visible”.

Simultaneously, “Pro Chemnitz” concentrate on the “us” component of the movement's collective identity. By situating their own protests in the context of the demonstrations in East Germany in 1989, they apply the dimension of urgency of motivational frames. There is talk of “Chemnitzers” (people who live in and “are from” Chemnitz), “Saxons”, “the people”; and the construction of this inner group is underpinned with images of demonstrators waving national, “black, red, and gold flags”, for which Höcke called in the days before:

The dignified image of a mourning community should not be disturbed by indiscipline. We therefore ask that you observe the following rules in keeping with the occasion: • Modest, preferably black clothing • No consumption of food or drink during the silent march • Smoking is also prohibited • Any kind of opinion expressed on clothing is prohibited (this also applies to political messages from the AfD), as is carrying posters and other advertising • Only black, red, and gold flags and white roses as a sign of mourning are permitted • We do not want extremists and violent offenders in our ranks—we are free citizens who want to express our grief for the dead and victims of illegal migration policy peacefully and respectfully! (Höcke, 28 August 2018)

Both collective actors heavily rely on a powerful German collective symbol: the peaceful revolution of the so-called Monday protesters in Eastern Germany in 1989 and the symbolic effect of the slogan “we are the people”, “the Volk”: “Goosebumps. We are the people. Thank you to everyone who was there.” (Pro Chemnitz, 07 September 2018, post 4). Besides, “German flags” (6 September 2018, post 3; 7 September 2018, posts 3, 4, 5; 8 on 9 September 2018) and banners carrying the slogan (e.g., 7 September 2018) are prevalent at their marches. Both flag and slogan were present at demonstrations in 1989 that contributed to pushing the government of the German Democratic Republic to open the country's borders and enter a process of democratization, and every adult in Eastern Germany can be expected to be able to relate to these events and its symbols. Yet, they have also been appropriated for far-right marches at the latest since the start of PEGIDA (Volk and Weisskircher, 2023) and “Querdenken” marches (Kalkstein and Dilling, 2024). By framing their protests in the city of Chemnitz in the sense of the 1989 political situation, “Pro Chemnitz” link their own mobilization attempts to the peaceful demonstrations to convince potential followers of their own “peaceful” intentions (see above). And “Querdenken 711” retweet: “we were many … we were peaceful!” (6 August, 2021). However, in connection with the other frames applied, this can also be interpreted as a wake-up call to “us” and a warning to “them”.

The different collective actors apply further symbols of peaceful resistance, primarily symbols of resistance against the National Socialist regime of 1933. Höcke calls for people to wear “a white rose” (28 August 2018) as a sign of mourning; the photograph of a white rose also appears in his very first post on *Chemnitz*. The white rose as a symbol is ambiguous enough to make ignorant readers of Höcke's believe that he is only interested in “peaceful” and “solemn” “mourning”, as he claims. However, his choice—the white rose as a symbol of the resistance group around Sophie and Hans Scholl against the National Socialist regime in Germany in Munich—in combination with “black, red, and gold flags” is also provocative and polarizing. It thus serves his framing of an alleged gap between the government and “the people”.

Three years after *Chemnitz*, a white rose appears in a “Querdenken” post. “Querdenken 30” object to the search of a judge's home: “The government shivers. Reverently before a flower …! […] Symbol for the end of the rule of law.” (1 May 2021). Here again, the flower is presented as a pure, innocent, harmless, and peaceful form of resistance. It has not been only picked up by the far right but also by “anti-vaxxers” (Fox, 2021), and both movements have increasingly misused comparisons with the Shoah and applied antisemitic conspiracies to mobilize supporters (Fox, 2021). Not as part of the online communication analyzed here, but in a broader context, “Querdenken” groups adopted the yellow star that the Jewish population of the Third Reich and occupied territories had to wear as a means of discrimination from 1941 on. “Querdenken” protesters replace “Jew” by “not vaccinated” to express their feeling of being discriminated against as a social group (Balandat, 2021). “Querdenken” groups easily overlap and merge with anti-vaxxer groups, who had already existed before COVID-19 restrictions were put into place in Germany and elsewhere (Bruni, 2020; Nachtwey and Frey, 2021). “Querdenken 711” explicitly show their similarity in thinking by adapting anti-vaxxers' “#SafeTheChildren” hashtag (e.g., 19 August 2021 post 2) or by posting templates for letters with the intention of applying for exceptions to prevent their children from having to wear masks (20 August 2021, post 2).

In both case studies, the collective actors use symbols of peaceful resistance from German history and reinterpret them for their own purposes of mobilizing potential followers who might still be hesitant regarding the aims of the protests and not yet willing to march with them. In both cases, these protests were not as “peaceful” as announced; the “funeral march” (see above) was shut down by police in the end. Höcke does not seek the blame for this with his supporters but states: “We had to stop our funeral march. This government is no longer able to protect us” (2 September 2018). In a similar vein, “Querdenken 30” try to show their power to their followers after the shut-down of one of their protests by claiming that “despite massive police presence, (the protesters) were not deterred and showed that power really does come from the people. […] Our focus is […] still on the restoration of fundamental rights” (22 August 2021). “Pro Chemnitz” proclaim a week into their mobilization after a successful protest: “The city is ours!” (7 September 2018, post 5). Ultimately, these efforts are about gaining space—or rather, about displacing those who are read as opponents or “others” (Jäkel, 2021).

## 6 Discussion

For this study, I was interested in how far-right actors in two case studies—*Chemnitz* and “Querdenken”—construct a collective identity through framing in their online communication on Facebook. I applied Benford and Snow's framing approach (2000) and added further dimensions (the analysis of emotions, the process of identity-construction) to my analysis.

The collective actors in the two case studies present specific identifications of problems and attribute responsibility to specific groups or people to blame in the diagnostic dimension of their framing. Regarding prognostic frames, I was able to identify framing of a possible solution to the problems the collective actors identified; yet they did not present a concrete plan to reach that solution. Indeed, only a very blurry suggestion is made—“resistance”—which leaves to their followers what to make of it in practice.

To a much larger extent, the collective actors at hand follow a dramaturgy of adapting the master frames. Mainly consisting of enemy constructions (“them”) and conspiracy myths, these master frames establish a connection between frames during the two discursive events at hand, as well as across the two case studies. In line with this finding, across the posts of any collective actor, one finds little variation, but specification of enemy images applied. Master frames across the two case studies become interrelated through the heavy use of conspiracy myths, i.e., the collective actors soon go beyond the respective trigger event and start referring to more abstract categories of “enemies” allegedly cooperating in a conspiracy. As shown, Höcke's chronological application of enemy constructions in his online communication serves the gradual introduction of alleged enemies (Schmidt-Kleinert, 2021) to a great extent. The other collective actors at hand do not adapt Höcke's chronological dramaturgy but they do adapt his framing of constructing collective “others” or out-groups (“them”).

Table 4 illustrates clearly that nearly every frame identified in the analysis is a master frame—if not across the discursive event, thus at least across actors that contributed to the same discursive event. It is interesting to state in this context that the collective actors at hand extensively pay attention to updating master frames that are well know from far-right discursive practices in Germany and transnationally. Examples are the emphasis on a constant state of “crisis” and the application of oppositional frames to construct the out-groups of “the media”, “the government”, or “puppet masters”.

**Table 4:** Overview of the application of frames by the collective actors under discussion.

**Public group name**	**Frames applied**	**Master frames applied**
Björn Höcke		**Diagnostic master frame:** “crisis” and the “government's mishandling” **Diagnostic master frame:** “cartel media” **Diagnostic master frame:** “puppet masters” and “marionettes” **Prognostic master frame:** “peaceful resistance” and with this collective action frame and in-group (“us”)
“Pro Chemnitz”	**Diagnostic frame:** “War of Cultures” (Motivational frame: urgency)	**Diagnostic master frame:** “crisis” and the “government's mishandling” **Diagnostic master frame:** “cartel media” **Diagnostic master frame:** “puppet masters” and “marionettes” **Prognostic master frame:** “peaceful resistance” and with this collective action frame and in-group (“us”)
“Querdenken 030”		**Diagnostic master frame:** “rule of law” **Diagnostic master frame:** “crisis” and the “government's mishandling” **Diagnostic master frame:** “cartel media” **Diagnostic master frame:** “puppet masters” and “marionettes” **Prognostic master frame:** “peaceful resistance” and with this collective action frame and in-group (“us”)
“Querdenken 711”		**Diagnostic master frame:** “rule of law” **Diagnostic master frame:** “crisis” and the “government's mishandling” **Diagnostic master frame:** “cartel media” **Diagnostic master frame:** “puppet masters” and “marionettes” **Prognostic master frame:** “peaceful resistance” and with this collective action frame and in-group (“us”)

It is also interesting to note that the analysis shows that the collective actors under discussion apply exactly one prognostic frame, the master frame of “peaceful resistance”. Besides, in my data, only “Pro Chemnitz” touched the motivational task of framing. A possible interpretation of these findings is that the collective actors as representative of the far right in Germany have grown confident that their supporters and potential followers have understood their message about who is to blame for the various “crises” they identify who are the' enemies” of their “völkisch”-read in-group, and consecutively, what their followers have to do about it within the range of “peaceful resistance” that has been applied by far-right groups at the various discursive events in the recent past. In this context, the latter term is still blurry enough to also attract new followers to their ranks. On the contrary, it is sufficient to update supporters on the “current crisis” and the out-group(s) responsible that needs resistance. But supporters do not have to be motivated but just to be kept on the level of action, as there is constant and sufficient motivation through the constant repetition of a state of “crisis” by various local, national and transnational collective actors within the far-right spectrum (in Germany and transnationally) that construct the respective discursive events.

In this context, analysis of communication on *Chemnitz* demonstrates the process of construction over time. In “Querdenken” posts, on the other hand, the category of “us” is not filled with content at all, as if it had become obvious by then to actual and potential members of the social movement and their opponents who belongs. Interpretation of the empirical findings suggests that the antagonism of *Chemnitz* has in “Querdenken” become a boundary that separates those within the social movement who believe in the “völkisch”-primordial constitution of their collective (Eisenstadt and Giesen 1991), and those who do not present an existential threat.

Against this background, the collective actors extensively use emotions in their framing, mainly applying negative emotions of grief, fear (of loss, existential threat) and anger or rage (against “the media”, “the elites”). The only positive emotion referred to in the posts is (national) pride and addresses a feature of the movements” collective identity. The “them”-dimension discussed in length by the collective actors at hand serve as the basis for the positive dimension (“us”) of their movements” collective identity, and both, Höcke and “Pro Chemnitz”, construct the “us” in direct opposition. Yet, they apply identity frames to a much lesser degree and more subtly than oppositional frames. In other words, the far-right collective actors at hand need the out-groups they discursively construct to construct a positive notion of the movement's self against them. These constructions go hand in hand with the heavy use of negative emotions, such as fear and anger. In this regard, it is not surprising that prognostic framing is hardly touched, and the collective actors apply identity frames only after they had made absolutely clear who the “enemy” is.

To sum up, I was able to show that the discursive images of out-groups constructed, against which the far-right collective actors at hand constructed a positive in-group, have entered the collectively shared knowledge of far-right social movement(s) in Germany as master frames. I showed how the collective actors at hand helped to (re-)construct and to update those master frames. Moreover, my analysis highlights the central role of conspiracy myths for collective identity-building and that they, too, have become collectively shared knowledge (Berger and Luckmann, 1966) across the two discursive events. Previous empirical studies, in contrast, focused on single discursive events or far-right actors and therefore lacked to show a process of frame application or the development of master frames over time and across discursive events. A further innovative aspect of the study at hand is that my analysis stresses the analytical worth to extend Benford and Snow's approach to frame analysis with foci on emotions and the process of identity construction. Further, these empirical findings contribute to (Castelli Gattinara and Pirro 2019) argument of thinking of the far right as one social movement. To further elaborate on their argument, a next necessary step is further comparative analysis of master frames applied by far-right collective actors in other timely discursive events in Germany and transnationally.

## Data Availability

The raw data supporting the conclusions of this article will be made available by the authors, without undue reservation.

## Footnotes

1 Members of these groups use this label to express an idea of ‘thinking against the mainstream'.

2 “PEGIDA” stands for “Patriotic Europeans Against the Islamization of the West”. It is a German far-right anti-Islam movement.

3 “QAnon” is a conspiracy myth that started in 2014 on the far-right image-board websites 4chan and 8chan. It uses modern antisemitic and anti-Jewish stereotypes (e.g., blood drinking and satanism), among others, as well as accusations of child sexual abuse to discredit (representatives of) the Democratic party in the USA (Lipowsky, 2020).

4 I initially observed the public Facebook groups connected to the following far-right collective actors: Björn Höcke (https://www.facebook.com/Bjoern.Hoecke.AfD/), ‘Bürgerbewegung Pro Chemnitz' (https://de-de.facebook.com/prochemnitz), AfD Chemnitz (https://de-de.facebook.com/AfD.Chemnitz/), Nico Köhler (https://de-de.facebook.com/nico.koehler.chemnitz), Martin Frohnmeier (https://de-de.facebook.com/frohnmaier). I used a fake account to have reading access but did not subscribe to the respective public group.

5 Caiani (2023) applied a similar two-step combination of discourse and frame analysis.

## References

[B1] Ahmed R. Pisoiu D. (2021). Uniting the far right: how the far-right extremist, New Right, and populist frames overlap on Twitter – a German case study. Eur. Soc. 23, 232–254. doi: 10.1080/14616696.2020.1818112

[B2] Balandat F. (2021). Antisemitismus auf Querdenker-Demos: Soll man das Tragen des “Ungeimpft”-Sterns verbieten?. Interview with Nicole Dittner. Deutschlandfunk Kultur, May 2021. Available online at: https://www.deutschlandfunkkultur.de/antisemitismus-auf-querdenker-demos-soll-man-das-tragen-des-100.html (Accessed October 5, 2025).

[B3] Benford R. D. Hunt S. A. (1992). Dramaturgy and social movements: the social construction and communication of power. Sociol. Inq. 62, 36–55. doi: 10.1111/j.1475-682X.1992.tb00182.x

[B4] Benford R. D. Snow D. A. (2000). Framing processes and social movements: an overview and assessment. Annu. Rev. Sociol. 26, 611–639. doi: 10.1146/annurev.soc.26.1.611

[B5] Berger P. L. Luckmann T. (1966). The Social Construction of Reality: A Treatise in the Sociology of Knowledge. Garden City, NY: Doubleday.

[B6] Bleakley P. (2023). Panic, pizza and mainstreaming the alt-right: A social media analysis of Pizzagate and the rise of the QAnon conspiracy. Curr. Sociol. 71, 509–525. doi: 10.1177/00113921211034896

[B7] Brichzin J. Laux H. Bohmann U. (2022). Risikodemokratie: Chemnitz zwischen rechtsradikalem Brennpunkt und europäischer Kulturhauptstadt. Bielefeld: transcript. German.

[B8] Bruni E. (2020). Institutional Theory and Discourse Analysis: An Empirical Investigation of the Rhetoric of Anti Vaccination Movement. DISCOURSEVAX Project Fact Sheet, H2020 CORDIS European Commission. Available online at: https://cordis.europa.eu/project/id/842019 (Accessed October 5, 2025).

[B9] Bundesamt für Verfassungsschutz R. R. (2025). Verfassungsschutzrelevante Delegitimierung des Staates. Available online at: https://www.verfassungsschutz.de/DE/themen/verfassungsschutzrelevante-delegitimierung-des-staates/verfassungsschutzrelevante-delegitimierung-des-staates_node.html (Accessed October 5, 2025).

[B10] Caiani M. (2023). Framing and social movements. Discourse Stud. 25, 195–209. doi: 10.1177/14614456231154734

[B11] Caiani M. della Porta D. (2011). The elitist populism of the extreme right: a frame analysis of extreme right-wing discourses in Italy and Germany. Acta Polit. 46, 180–202. doi: 10.1057/ap.2010.28

[B12] Castelli Gattinara P. Pirro A. L. (2019). The far right as social movement. Eur. Soc. 21, 447–462. doi: 10.1080/14616696.2018.1494301

[B13] Closmann J. S. (2020). Unsocial Web: Zur Virtualität von rechten Bewegungen. Forum Demokratieforschung Working Paper Series No. 16. Marburg: Philipps-Universität Marburg. Available online at: https://www.uni-marburg.de/de/fb03/politikwissenschaft/fachgebiete/brd/working-paper/working-paper-no-16-unsocial-web-zur-virtualitat-rechter-bewegungen-2020.pdf (Accessed October 5, 2025).

[B14] Daphi P. (2011). Soziale Bewegungen und kollektive Identität. Forschungsjournal Soz. Beweg. 24, 13–27. German. doi: 10.1515/fjsb-2011-0404

[B15] Der Tagesspiegel Online (2021). Facebook löscht Kanäle und Gruppen der “Querdenker”. Berlin: Der Tagesspiegel. German.

[B16] Diani M. (1992). The concept of social movement. Sociol. Rev. 40, 1–25. doi: 10.1111/j.1467-954X.1992.tb02943.x

[B17] Eisenstadt S. N. (1998). The construction of collective identities: some analytical and comparative indications. Eur. J. Soc. Theory 1, 229–254. doi: 10.1177/136843198001002008

[B18] Evans S. K. Pearce K. E. Vitak J. Treem J. W. (2017). Explicating affordances: a conceptual framework for understanding affordances in communication research. J. Comput. Mediat. Commun. 22, 35–52. doi: 10.1111/jcc4.12180

[B19] Evolvi G. (2019). Emotional politics, Islamophobic tweets: the Hashtags #Brexit and #chiudiamoiporti. Partecip. Confl. 12, 871–897. doi: 10.1285/i20356609v12i3p871

[B20] Flesher Fominaya C. (2010). Collective identity in social movements: central concepts and debates. Sociol. Compass 4, 393–404. doi: 10.1111/j.1751-9020.2010.00287.x

[B21] Fox M. (2021). Anti-Vaxxers Have Stolen an Anti-Nazi Group's Identity. The Forward. Available online at: https://forward.com/news/474596/anti-vaxxers-have-stolen-an-anti-nazi-groups-identity/ (Accessed October 5, 2025).

[B22] Gagnon A. (2020). Far-right framing processes on social media: the case of the Canadian and Quebec Chapters of Soldiers of Odin. Can. Rev. Sociol. 57, 356–378. doi: 10.1111/cars.12291 32779365

[B23] Glaser B. G. Strauss A. L. (2010). Grounded Theory: Strategien qualitativer Forschung. 3rd Edn. Bern: Huber. German.

[B24] Goffman E. (1959). The Presentation of Self in Everyday Life. Garden City, NY: Doubleday.

[B25] Götschenberg M. (2022). Gefährliche Mischung. tagesschau online. Available online at: https://www.tagesschau.de/inland/innenpolitik/reichsbuerger-szene-101.html (Accessed October 5, 2025).

[B26] Jacobs K. Spierings N. (2019). A populist paradise? Examining populists' Twitter adoption and use. Inform. Commun. Soc. 22, 1681–1696. doi: 10.1080/1369118X.2018.1449883

[B27] Jäger S. (2015). Kritische Diskursanalyse: Eine Einführung, 7th Edn. Münster: Unrast-Verlag. German.

[B28] Jäkel L. (2021). “Gelände- und Machtgewinne rechter Akteur^*^innen in der virtuellen und realen Welt,” in Mobilisierungsstrategien kollektiver Akteure der extremen Rechten und des salafistischen Dschihadismus, Eds. U. Birsl, J. Junk, M. Kahl, and R. Pelzer (Opladen: Barbara Budrich), 137–158. German.

[B29] Kakavand A. E. (2024). Far-right social media communication in the light of technology affordances: a systematic literature review. Ann. Int. Commun. Assoc. 48, 37–56. doi: 10.1080/23808985.2023.2280824

[B30] Kalkstein F. Dilling M. (2024). ‘1989' als Mythos – Apokalypse als Neuanfang: eine tiefenhermeneutische Fallanalyse der apokalyptischen Narrative und der Wendebezüge auf den Corona-Protesten. ZRex Z. Rechtsextremismusforsch. 4, 169–189. German. doi: 10.3224/zrex.v4i2.03

[B31] Kelly M. DiBranco M. DeCook J. R. (2021). Misogynist Incels and Male Supremacism: Overview and Recommendations for Addressing the Threat of Male Supremacist Violence. Washington, DC: New America.

[B32] Klein O. Muis J. (2019). Online discontent: comparing Western European far-right groups on Facebook. Eur. Soc. 21, 540–562. doi: 10.1080/14616696.2018.1494293

[B33] Knappertsbusch F. (2016). Antiamerikanismus in Deutschland: Über die Funktion von Amerikabildern in nationalistischer und ethnozentrischer Rhetorik. Bielefeld: transcript. German.

[B34] Krämer B. (2017). Populist online practices: the function of the Internet in right-wing populism. Inform. Commun. Soc. 20, 1293–1309. doi: 10.1080/1369118X.2017.1328520

[B35] Langer A. (2022). “Deep State, child sacrifices, and the ‘Plandemic': the historical background of antisemitic tropes within the QAnon movement,” in Antisemitism on Social Media, Eds. M. Hübscher and S. von Mering (London/New York: Routledge) 18–34.

[B36] Lindekilde L. (2014). “Discourse and frame analysis: in-depth analysis of qualitative data in social movement research,” in Methodological Practices in Social Movement Research, Ed. D. Della Porta (Oxford: Oxford University Press) 195–227.

[B37] Lipowsky J. (2020). QAnon Conspiracies Mirror Historic Anti-Semitism. Jewish Standard. Available online at: https://jewishstandard.timesofisrael.com/qanon-conspiracies-mirror-historic-anti-semitism/ (Accessed October 5, 2025).

[B38] Melucci A. (1995). “Social movements and the transformation of collective identity,” in Social Movements and Culture, Eds. H. Johnston and B. Klandermans (Minneapolis, MN: University of Minnesota Press) 41–63.

[B39] Merrill S. Åkerlund M. (2018). Standing up for Sweden? The racist discourses, architectures and affordances of an anti-immigration Facebook group. *J. Comput.-Mediat. Commun*. 23, 332–353. doi: 10.1093/jcmc/zmy018

[B40] Moffitt B. (2015). How to perform crisis: a model for understanding the key role of crisis in contemporary populism. Gov. Oppos. 50, 189–217. doi: 10.1017/gov.2014.13

[B41] Mudde C. Kaltwasser C. R. (2017). Populism: A Very Short Introduction. Oxford: Oxford University Press.

[B42] Nachtwey O. Frey N. (2021). Quellen des “Querdenkertums”: Eine politische Soziologie der Corona-Proteste in Baden-Württemberg. Heinrich-Böll-Stiftung Baden-Württemberg Report. Stuttgart: Heinrich-Böll-Stiftung Baden-Württemberg. Available online at: https://www.boell-bw.de/sites/default/files/2021-11/Studie_Quellen%20des%20Querdenkertums.pdf (Accessed October 5, 2025).

[B43] Pfahl-Traughber A. (2019). Was die “Neue Rechte” ist – und was nicht. Bundeszentrale für politische Bildung (bpb). Available online at: https://www.bpb.de/themen/rechtsextremismus/dossier-rechtsextremismus/284268/was-die-neue-rechte-ist-und-was-nicht/ (Accessed October 5, 2025).

[B44] Polletta F. Jasper J. M. (2001). Collective identity and social movements. Annu. Rev. Sociol. 27, 283–305. doi: 10.1146/annurev.soc.27.1.283

[B45] Polta P. (2023). Antisemitismus und Antifeminismus in Covid-19-Verschwörungsmythen. ZRex Z. Rechtsextremismusforsch. 3, 68–82. German. doi: 10.3224/zrex.v3i1.05

[B46] Raschke J. (1985). Soziale Bewegungen: Ein historisch-systematischer Grundriß. Frankfurt am Main: Campus. German.

[B47] Rucht D. (2023). Social Movements. Cambridge: Polity Press.

[B48] Salzborn S. (2020). Globaler Antisemitismus: Eine Spurensuche in den Abgründen der Moderne, 2nd Edn. Weinheim: Juventa. German.

[B49] Schilk F. (2024). Die Erzählgemeinschaft der Neuen Rechten: Zur politischen Soziologie konservativer Krisennarrative. Bielefeld: transcript. German.

[B50] Schmidt-Kleinert A. (2021). “Der “absolute' Feind: Feindbildkonstruktionen in den sozialen Medien zum Gewaltereignis Chemnitz”,” in Inszenieren und Mobilisieren: Rechte und islamistische Akteure digital und analog, Eds. U. Birsl, J. Junk, M. Kahl, and R. Pelzer (Opladen: Barbara Budrich), 109–136. German.

[B51] Soeffner H.-G. (1992). Die Ordnung der Rituale. Die Auslegung des Alltags 2. Frankfurt/M.: Suhrkamp.

[B52] Stoppt die Rechten R. R. (2018). Die feigen Gestalten da oben. September 13, 2018. Available online at: https://www.stopptdierechten.at/2018/09/13/die-feigen-gestalten-da-oben/ (Accessed October 5, 2025).

[B53] Sundermeyer O. (2020). Rechtsextremisten und “Querdenken 711”: “Man will gemeinsam die Regierung stürzen”. Interview with S. Meschkat. Deutschlandfunk, November 16, 2020. Available online at: https://www.deutschlandfunk.de/rechtsextremisten-und-querdenken-711-man-will-gemeinsam-die-100.html (Accessed October 5, 2025).

[B54] Swart W. (1995). The League of Nations and the Irish Question: master frames, cycles of protest, and ‘Master Frame Alignment'. Sociol. Q. 36, 465–481. doi: 10.1111/j.1533-8525.1995.tb00448.x

[B55] Virchow F. Häusler A. Döring M. (2020). Pandemie-Leugnung und extreme Rechte in Nordrhein-Westfalen. CoRE NRW Kurzgutachten. Düsseldorf: CoRE NRW. Available online at: https://www.ssoar.info/ssoar/handle/document/88176 (Accessed October 5, 2025).

[B56] Volk S. (2023). Resisting ‘leftist dictatorship'? Memory politics and collective action framing in populist far-right street protest. Eur. Polit. Soc. 24, 535–551. doi: 10.1080/23745118.2022.2058756

[B57] Volk S. Weisskircher M. (2023). Defending democracy against the ‘Corona Dictatorship'? Far-right PEGIDA during the COVID-19 pandemic. Soc. Mov. Stud. 23, 719–737. doi: 10.1080/14742837.2023.2171385

[B58] Weiß V. (2017). Die autoritäre Revolte: Die Neue Rechte und der Untergang des Abendlandes. Stuttgart: Klett-Cotta. German.

[B59] ZEIT Archiv R. R. (1982). Was sich deutsche Professoren bei der Unterschrift unter das “Heidelberger Manifest” dachten. February 5, 1982. Die ZEIT. Available online at: https://www.zeit.de/1982/06/rassistische-klaenge (Accessed October 5, 2025).

